# The Role of Micro-Ultrasound in Prostate Cancer Detection

**DOI:** 10.1007/s11934-026-01371-y

**Published:** 2026-10-09

**Authors:** Navid Jabalameli, Feres Camargo Maluf, Fatema Bhinderwala, Thomas J. Polascik, Soroush Rais-Bahrami

**Affiliations:** 1 Department of Urology, Wake Forest University School of Medicine, Winston-Salem, NC USA; 2 Department of Urology, University of Alabama at Birmingham Heersink School of Medicine, Birmingham, AL USA; 3 Department of Radiology, Wake Forest University School of Medicine, Winston-Salem, NC USA; 4 Atrium Health Wake Forest Baptist Comprehensive Cancer Center, Wake Forest University School of Medicine, Winston-Salem, NC USA; 5 Department of Cancer Medicine, Wake Forest University School of Medicine, Winston-Salem, NC USA

**Keywords:** Micro-ultrasound, Prostate cancer, Targeted prostate biopsy, Multiparametric MRI, PRI-MUS

## Abstract

**Purpose of Review:**

To synthesize current evidence on the role of 29-MHz micro-ultrasound (microUS) in prostate cancer (PCa) detection and to define its position relative to multiparametric MRI (mpMRI) across the diagnostic pathway. We conducted a narrative review of prospective, comparative, and randomized studies, with clinically significant prostate cancer (csPCa) detection as the primary endpoint.

**Recent Findings:**

MicroUS provides ~ 70-µm axial resolution and enables real-time PRI-MUS–based risk stratification. Across head-to-head studies, microUS achieves diagnostic performance comparable to mpMRI. Evidence supports utility in biopsy-naïve patients, selected MRI-negative men with persistent clinical suspicion, repeat-biopsy settings, active surveillance, and local tumor staging, while combined mpMRI–microUS approaches have been shown to maximize yield of csPCa detection. Key limitations include operator dependence, interobserver variability, lack of validated biopsy-avoidance thresholds, and reduced sensitivity in anterior lesions.

**Summary:**

MicroUS is a practical, high-resolution platform that complements mpMRI and may enhance accessibility, efficiency, and precision in prostate cancer care.

## Introduction

Prostate cancer (PCa) is the most commonly diagnosed non-cutaneous malignancy in men, with approximately 1.5 million new cases worldwide annually and an estimated 314,000 new cases and 35,800 deaths projected in the United States in 2025 [1, 2]. While Prostate-specific antigen (PSA)-based screening has reduced the incidence of advanced disease, it lacks specificity for clinically significant prostate cancer (csPCa), making differentiation between indolent and clinically significant disease a persistent challenge [3, 4]. Overdiagnosis can lead to unnecessary treatment with urinary, sexual, and bowel morbidity, whereas underdetection of csPCa may delay management and compromise oncologic outcomes [4, 5]. 

The standard 12-core systematic biopsy with conventional transrectal ultrasound (TRUS) relies on non-targeted sampling, contributing to both under-detection of significant tumors and overdiagnosis of indolent disease. TRUS demonstrates limited lesion discrimination (positive predictive value [PPV] 18–42%) and may miss up to 48% of csPCa cases [6–8]. 

Multiparametric MRI (mpMRI) has improved PCa diagnosis by enabling lesion localization and characterization through the Prostate Imaging Reporting and Data System (PI-RADS) and is recommended by major international guidelines for pre-biopsy assessment and biopsy targeting [9–11]. In the PROMIS trial, mpMRI used as a triage test could have avoided 27% of primary biopsies while detecting 18% more csPCa than TRUS-guided biopsy [12]. MRI–ultrasound fusion biopsy further improves risk stratification, increasing high-risk cancer detection and reducing low-risk disease detection versus standard extended-sextant biopsy [13, 14]. However, mpMRI remains limited by suboptimal sensitivity (≥ 15% miss rate for Grade Group [GG] ≥ 2 disease), interobserver variability, and workflow complexity [15, 16]. At ~$2,550 per scan (estimated average charge in the US), universal pre-biopsy adoption would amount to an estimated $3 billion annually for the 1.0–1.2 million men undergoing biopsy, while contraindications and limited availability further restrict use in resource-constrained settings [17]. 

29-MHz micro-ultrasound (microUS), introduced in the mid-2010s, provides real-time, high-resolution visualization of prostate microarchitecture in an office-based setting [18]. It has progressed from feasibility studies to multicenter prospective and randomized trials, with meta-analyses demonstrating csPCa detection comparable to mpMRI alongside potential advantages in cost, accessibility, and workflow integration [15, 19–22]. 

This narrative review evaluates the current evidence supporting microUS in the PCa diagnostic pathway, with particular focus on its potential to improve in-office workflow efficiency, real-time lesion visualization and direct targeting, while reducing reliance on fusion-based systems, resource-intensive imaging, costs, and unnecessary biopsies.

## Technical Principles of Micro-Ultrasound

MicroUS evolved from preclinical high-frequency ultrasound platforms developed from the late 1980s through the 2000s. Clinical application to the prostate was first reported in 2014, through an in-vivo imaging–pathology correlation study using whole-mount radical prostatectomy specimens, which demonstrated improved sensitivity and specificity over conventional ultrasound [23–26]. 

The principal platform, ExactVu™ (Exact Imaging, Markham, ON, Canada), uses the EV29L side-fire transducer (29 MHz, 512-element linear array, ~ 90 μm spacing), achieving ~ 70 μm axial resolution, sufficient to resolve prostatic ductal architecture (~ 100 μm), where structural disruption correlates with histopathologic grade [15, 27]. Distance-based beamforming and advanced signal processing provide a threefold resolution improvement compared to conventional TRUS (6–9 MHz; ~200 μm) while preserving ~ 5–6 cm penetration, adequate for the peripheral zone, where ~ 70% of prostate cancers arise [18, 28, 29]. As a modality that relies on tissue structure, microUS is similar to T2-weighted MRI but lacks functional sequences such as diffusion-weighted (DWI) and dynamic contrast-enhanced (DCE) imaging [6, 18]. The platform also supports software-assisted MRI–microUS fusion (FusionVu™), enabling combined targeting without prostate contouring [20, 26]. 

### PRI-MUS Risk Stratification

The Prostate Risk Identification using Micro-Ultrasound (PRI-MUS) system provides a standardized 5-point framework for real-time lesion characterization based on echogenicity, ductal architecture, and margins, analogous to PI-RADS; scores of 3 or higher are considered suspicious for csPCa [18]. Developed from cine loops of 400 biopsies and validated in 100 pathology-blinded cines, PRI-MUS initially showed 80% sensitivity and 37% specificity for csPCa, with each incremental increase in score associated with ~ 10% higher csPCa probability and improved performance for higher-grade disease (Area Under the Curve [AUC] 74%) [18] (Table 1).

**Table 1:** Comparison of the prostate risk identification using micro-ultrasound (PRI-MUS) protocol and the prostate imaging–reporting and data system version 2.1 (PI-RADS v2.1) [11, 18]

Score (csPCa likelihood)	Micro-ultrasound (microUS) PRI-MUS protocol	Multiparametric MRI (mpMRI) PI-RADS v2.1
Score 1 Very low likelihood	Normal-appearing prostate with regular, evenly spaced ducts (“Swiss-cheese” pattern); no heterogeneity and no bright echoes.	**PZ (DWI-dominant)**: No abnormality on ADC and high b-value DWI (DWI score 1); uniform hyperintense (normal) signal on T2W imaging. **TZ (T2W-dominant)**: Normal-appearing TZ (rare) or a round, completely encapsulated nodule (“typical nodule”).
Score 2 Low likelihood	Hyperechoic tissue with or without ductal patches; possible ectatic ducts or cysts (“starry sky” appearance).	**PZ (DWI-dominant)**: Linear or wedge-shaped hypointensity on ADC and/or linear or wedge-shaped hyperintensity on high b-value DWI (DWI score 2). **TZ (T2W-dominant)**: Mostly encapsulated nodule, or homogeneous circumscribed nodule without encapsulation (“atypical nodule”), or homogeneous mildly hypointense area between nodules. A DWI score ≥ 4 upgrades a score-2 TZ lesion to category 3.
Score 3 Intermediate / indeterminate	Mild heterogeneity or bright echoes within hyperechoic tissue.	**PZ (DWI-dominant)**: Focal (discrete and different from the background) hypointensity on ADC and/or focal hyperintensity on high b-value DWI; may be markedly hypointense on ADC or markedly hyperintense on high b-value DWI, but not both (DWI score 3). A positive DCE upgrades a score-3 PZ lesion to category 4. **TZ (T2W-dominant)**: Heterogeneous signal intensity with obscured margins; includes lesions that do not qualify as 2, 4, or 5. A DWI score of 5 upgrades a score-3 TZ lesion to category 4.
Score 4 High likelihood	Mixed-echo pattern with ductal anarchy; smudgy, mottled, or cauliflower-like heterogeneity; bright echoes suggestive of comedonecrosis.	**PZ (DWI-dominant)**: Focal markedly hypointense on ADC and markedly hyperintense on high b-value DWI; <1.5 cm in greatest dimension (DWI score 4). **TZ (T2W-dominant)**: Lenticular or non-circumscribed, homogeneous, moderately hypointense, and < 1.5 cm in greatest dimension.
Score 5 Very high likelihood	Irregular shadowing originating from within the prostate (not from the prostate border), or mixed-echo lesions distorting the prostate and/or peripheral-zone border.	**PZ and TZ**: As Score 4 but ≥ 1.5 cm in greatest dimension, or definite EPE / invasive behavior.

Each score reflects an increasing likelihood of clinically significant prostate cancer (csPCa; Grade Group ≥ 2). The two systems are independently developed five-tier ordinal risk scales; the side-by-side alignment denotes parallel risk strata of increasing csPCa likelihood and does not imply a validated score-to-score equivalence. PI-RADS v2.1 applies separate dominant-sequence criteria for the peripheral zone (PZ; DWI/ADC) and the transition zone (TZ; T2W) and uses a 1.5 cm size threshold to distinguish categories 4 and 5; the original PRI-MUS protocol does not incorporate lesion size and was developed and validated primarily in the peripheral zone. Abbreviations: *ADC* apparent diffusion coefficient, *DCE* dynamic contrast-enhanced imaging, *DWI* diffusion-weighted imaging, *EPE* extraprostatic extension, *PZ* peripheral zone, *T2W* T2-weighted imaging, *TZ* transition zone.

Subsequent studies confirmed a strong correlation between PRI-MUS scores and disease aggressiveness: scores ≥ 3 identified 68% of GG2 and all GG3 tumors in one prospective cohort [30]. In external validation within the OPTIMUM trial, no ≥GG2 cancers were observed in PRI-MUS 1 lesions; PRI-MUS 1–2 demonstrated an 83% negative predictive value (NPV), and 86% of PRI-MUS 5 lesions harbored ≥GG2 disease, with fewer than 10% limited to GG1 [31]. 

PRI-MUS v2 expanded assessment to anterior and whole-gland regions underrepresented in the original protocol, and a later refinement introduced sagittal length ≥ 2 cm as a quantitative criterion for PRI-MUS 5, raising the PPV of PRI-MUS 4 and 5 lesions from 50.9% to 60.3% and 85.5% to 88.4%, respectively, and adding the previously missing size-based discriminator [32, 33]. Observed discordance with PI-RADS suggests differences in tumor morphology and imaging visibility, supporting a complementary role alongside mpMRI [20, 34]. 

## Clinical Integration of MicroUS in Prostate Biopsy

Current AUA, EAU, and NCCN guidelines recommend mpMRI to enhance biopsy accuracy. However, MRI-targeted biopsy can miss up to ~ 20% of ≥GG2 cancers, supporting combined targeted and systematic approaches [9, 10, 35, 36]. 

Through PRI-MUS risk stratification, microUS enables dynamic needle guidance, adaptive sampling, and direct lesion targeting without fusion software [18, 28]. Compared with conventional TRUS, even when TRUS uses prior MRI information, microUS increases lesion conspicuity (67% versus 43%) while maintaining equivalent csPCa detection when lesions are visualized (58% versus 58%) [6, 37]. Other studies report high microUS lesion visibility (up to 90%) and substantial csPCa detection when MRI information is available [6, 28]. By operating independently of MRI fusion workflows, microUS avoids its workflow complexity, and software-registration errors, an advantage most relevant in the anterior gland, where probe-pressure deformation degrades fusion accuracy, and systematic TRUS undersamples csPCa [20, 38–41]. In a prospective study, replacing non-targeted systematic biopsy with microUS-targeted biopsy avoided 898 cores across 94 patients and reduced clinically insignificant PCa detection by 36%, while preserving csPCa detection comparable to combined approaches (systematic biopsy and MRI) [6]. Adding microUS to an established mpMRI-targeted workflow identified csPCa in ~ 13–17% of cases with negative MRI-targeted biopsy, indicating that it adds diagnostic value, complementing rather than duplicating MRI-directed sampling [42, 43]. PSA density (PSAD) may further refine risk stratification: a PSAD-modified microUS protocol (PRI-MUS ≥ 3 with PSAD ≥ 0.15 ng/mL² considered positive) demonstrated sensitivity 83.3%, specificity 59.1%, and NPV 89.7% for csPCa in a retrospective opportunistic screening cohort (median PSA 1.2 ng/mL), potentially reducing unnecessary biopsies and MRI utilization; however, these findings derive from a screening population and may not generalize to higher-risk biopsy cohorts, and performance in equivocal PRI-MUS 3 lesions remains uncertain [44–46]. 

The optimal number of targeted cores remains undefined: MRI-based protocols typically recommend 2–5 per lesion, whereas microUS may achieve effective sampling with fewer cores, reducing indolent-disease detection without compromising csPCa detection [6, 47]. 

The biopsy route also influences outcomes. Although transrectal biopsy (TRB) remains routine in many U.S. centers, transperineal biopsy (TPB) is increasingly preferred for lower infection risk and better anterior prostate access [48]. Detection rates are comparable (overall 72%, csPCa 48%), but TPB demonstrates a superior safety profile, with urosepsis mainly observed in TRB cohorts [41, 48, 49]. 

In summary, MicroUS integrates visualization, targeting, and sampling into a single real-time workflow, supporting its role as a complementary modality and, in selected clinical settings, as a primary platform for biopsy guidance.

## Diagnostic Performance

### Head-to-Head Comparison with mpMRI

The primary goal of PCa diagnosis is to accurately identify csPCa while minimizing unnecessary biopsies and overdiagnosis of indolent disease, thereby driving a shift from PSA-based systematic biopsy toward imaging-guided pathways, each with distinct trade-offs [9, 12, 50]. Although biparametric MRI (bp-MRI, which omits the dynamic contrast-enhanced sequence) demonstrated non-inferiority to mpMRI with reduced cost and faster acquisition in the PRIME trial, MRI of any form still requires a separate scanner visit, radiologist interpretation, and an asynchronous workflow that limits access in resource-constrained settings [17, 51, 52]. A multicenter prospective registry (*n* = 1,040) reported microUS sensitivity of 94% and NPV of 85% for csPCa, with only a 2% false-negative rate across all patients [15]. In the OPTIMUM randomized trial (*n* = 802), microUS-guided biopsy was noninferior to MRI/conventional-US fusion biopsy for csPCa detection (46% [160/347] versus 42.6% [141/331]; difference 3.5%, *p* < 0.001); adding MRI to microUS did not significantly improve detection over MRI fusion alone (46.9% versus 42.6%; difference 4.29%). Among the 226 patients imaged with both modalities, concordant positive imaging predicted csPCa in 73.6%, whereas detection was low when both were negative (8.8%). Seven csPCa cases were identified by MRI when microUS was negative, and 7 by microUS when MRI was negative, indicating symmetric discordant contributions [20]. A secondary analysis of OPTIMUM (347 patients; 4,590 cores; 322 lesions) externally validated PRI-MUS: at the per-core level, PRI-MUS ≥ 3 versus ≤ 2 yielded sensitivity 72%, specificity 74%, and NPV 93% for csPCa (AUC 0.78); at the per-lesion level, sensitivity rose to 92% with NPV 83%. Among 19 biopsied anterior high-risk lesions, csPCa was present in 58% [31]. 

Meta-analyses corroborate these findings. Sountoulides et al. (13 studies, *n* = 1,125) reported a csPCa detection ratio of 1.05 (95% CI 0.93–1.19) for microUS versus mpMRI-targeted biopsy, indicating statistical equivalence [21]. A subsequent diagnostic test accuracy meta-analysis confirmed high pooled sensitivity but consistently lower specificity, supporting microUS as a sensitivity-driven triage and rule-out tool rather than an independent rule-in confirmation [22]. 

In the head-to-head comparison study by Ghai et al., patient-level csPCa detection was comparable between microUS and mpMRI (35% versus 39%), with similar rates for GG1 (17% versus 16%) and adverse-pathology rates (14% versus 15%); however, mpMRI allowed significantly greater biopsy avoidance (34% versus 10%), likely reflecting microUS’s lower specificity and the absence of a validated biopsy-avoidance threshold analogous to PI-RADS ≤ 2 lesions [6]. At the lesion level, mpMRI identified 93 lesions and microUS 162, with 62 detected by both; concordant lesions yielded 58% csPCa, per-lesion PPV favored mpMRI (46% versus 27%), and each modality contributed a similar number of unique csPCa foci (microUS-only: 9; mpMRI-only: 8), supporting combined targeting to maximize csPCa yield while limiting detection of indolent disease [6, 43]. 

In prospective cohorts, microUS has shown performance broadly comparable to mpMRI (Table 2). Lughezzani et al. (*n* = 320, PI-RADS ≥ 3 required for inclusion, which may affect microUS performance relative to general practice) reported microUS sensitivity 89.7% and NPV 81.5% for csPCa detection, with combined targeted-plus-systematic biopsy detecting csPCa in 36.3% of patients. In patients with lesions seen on both modalities, location matched in 74% of cases, and PRI-MUS and PI-RADS scores were concordant in 61% of cases. MicroUS detected csPCa in 17.2% of mpMRI-negative patients while mpMRI detected csPCa in 12.1% of microUS-negative patients [43]. An updated series from the same group (*n* = 1,423) found microUS lesion visualization in 76% of patients with sensitivity 85% and NPV 79% for csPCa, 96% lesion concordance with mpMRI among the 804 patients with lesions on both modalities, and 25 csPCa cases detected exclusively by microUS targeting versus 4 by mpMRI targeting; PRI-MUS outperformed PI-RADS on direct receiver operating characteristic curve (ROC) analysis (AUC 0.69 versus 0.62, *p* < 0.0001), and adding imaging to clinical predictors raised the multivariable AUC from 0.72 to 0.79 [54].

**Table 2:** Prospective comparative studies evaluating micro-ultrasound versus MRI for the detection of clinically significant prostate cancer

Author (Year)	Study Design	Population / Setting	*N*	Biopsy Strategy	Overall csPCa Detection (%)	microUS Performance (%)	MRI Performance (%)	Key Findings
Klotz et al., [15]	Prospective multicenter registry	Mixed cohort (66% BN, 6.3% AS)	1040	microUS TBx + MRI TBx + SBx	39.5%	SE: 94 SP: 22 PPV: 44 NPV: 85	SE: 90 SP: 22 PPV: 43 NPV: 77	microUS SE superior to mpMRI with non-inferior SP for csPCa detection
Socarrás et al., [49]	Prospective single-center comparative study	Mixed cohort (~ 66% no prior biopsy) ¹	194	microUS TBx + MRI TBx + TP-SBx	41.8%	SE: 99.7 SP: 23.1 PPV: 46 NPV: 99.2 ⁷	SE: 84.3 SP: 18.8 PPV: 40.7 NPV: 64.5 ⁷	microUS improved relative SE/NPV vs. MRI; detected additional csPCa missed by MRI; complementary role
Lughezzani et al., [43]	Prospective single-center study	PI-RADS ≥ 3 required; 62.5% initial biopsy (biopsy-naïve) ²	320	microUS TBx + MRI TBx + SBx	36.3%	SE: 89.7 SP: 26 PPV: 40.8 NPV: 81.5	NR ²	microUS and MRI comparable csPCa detection; complementary, with additional yield from SBx
Wiemer et al., [42]	Prospective single-center study	Prior MRI+ (45% BN, 29% AS) ³	159	microUS TBx + MRI TBx + SBx	49.1%	SE: 95 SP: 15 PPV: 52 NPV: 75 ⁷	NR ³	Adding microUS to MRI improved csPCa detection; microUS identified additional cancer in 17% of MRI-TBx-negative cases
Ghai et al., [6]	Prospective single-center paired study	Biopsy-naïve	94	microUS TBx + MRI TBx + SBx	40% (microUS: 35%; MRI: 39%; combined: 40%)	NR⁴	NR ⁴	Comparable csPCa detection; MRI avoided more biopsies (34% vs. 10%); 67% of MRI lesions prospectively visible on microUS
Hofbauer et al., [19]	Prospective multicenter study	Prior +/− MRI (72% BN)	203	microUS TBx + MRI TBx + SBx	38.9%	csPCa detected: 73% SE/SP/PPV/NPV: NR ⁵	csPCa detected: 76% SE/SP/PPV/NPV: NR ⁵	microUS non-inferior to MRI for csPCa detection; detected 97% of csPCa cases identified by MRI-TBx
Maffei et al., [53]	Prospective single-center study	AS (ISUP 1)	100	microUS TBx + MRI TBx + SBx	34%	SE: 94.1 SP: 24.2 PPV: 39 NPV: 88.9	SE: 100 SP: 12.1 PPV: 37 NPV: 100	High SE/NPV comparable to MRI for csPCa reclassification in AS; microUS-negative could avoid 18% of biopsies with minimal missed upgrades
Kinnaird et al., [20]	Prospective multicenter randomized trial	Biopsy-naïve⁶	678⁶	3 arms: microUS TBx + SBx vs. MRI/US fusion TBx + SBx vs. microUS/MRI fusion TBx + SBx	microUS: 47.1% MRI/US:42.6% microUS/MRI:46.9%	NPV: 83.1% (PRI-MUS < 3)	NPV: 89.4% (PI-RADS < 3)	microUS non-inferior to MRI/US fusion for csPCa detection; concordant positive findings strongly predictive (73.6%); combined negative results associated with low csPCa detection (8.8%)

**¹** Socarrás et al. [49]: 65/194 patients (33.9%) had a prior biopsy; the biopsy-naïve proportion (~ 66%) is estimated from reported data.

**²** Lughezzani et al. [43]: 200/320 patients (62.5%) were in the initial-biopsy (biopsy-naïve) setting; all had ≥ 1 PI-RADS ≥ 3 lesion as an inclusion criterion. MRI diagnostic accuracy could not be assessed in this PI-RADS ≥ 3–only cohort and was not reported (NR); the study’s supplementary materials contain only a discordant-lesion subgroup analysis (Supplementary Table 2) and a microUS literature summary (Supplementary Table 3), neither of which reports MRI sensitivity, specificity, PPV, or NPV.

**³** Wiemer et al. [42]: Only mpMRI-positive patients were analyzed; MRI sensitivity/specificity could not be calculated (NR = not reportable, not missing).

**⁴** Ghai et al. [6]: Formal SE/SP not reported; per-modality detection rates are provided in the overall csPCa detection column.

**⁵** Hofbauer et al. [19]: The primary endpoint was the csPCa detection ratio, not absolute diagnostic accuracy; patient-level SE/SP/PPV/NPV were not reported (NR).

**⁶** Kinnaird et al. [20]-OPTIMUM trial: 802 patients randomized; 678 underwent biopsy. Three-arm design: microUS alone (*n* = 121), MRI/conventional US fusion (*n* = 331), microUS/MRI fusion (*n* = 226).

**⁷**Socarrás and Wiemer reported relative (not absolute) sensitivity and specificity: no independent reference standard (template mapping or prostatectomy) was used, so microUS contributed to its own reference, and the values are biased upward. For Wiemer, sensitivity (95%) and specificity (15%) are csPCa-specific, whereas PPV (52%) and NPV (75%) are for any prostate cancer.

Abbreviations: *AS* active surveillance, *BN* biopsy-naïve, *csPCa* clinically significant prostate cancer (ISUP grade group ≥2 in all studies), *microUS* micro-ultrasound, *MRI* multiparametric MRI, *NPV* negative predictive value, *NR* not reportable, *PPV* positive predictive value, *SBx* systematic biopsy, *SE* sensitivity, *SP* specificity, *TBx* targeted biopsy, *TP* transperineal.

Altogether, microUS achieves diagnostic performance comparable to mpMRI-targeted biopsy for csPCa detection across head-to-head trials and meta-analyses; mpMRI retains advantages in biopsy avoidance and asynchronous review, whereas microUS adds complementary detection where MRI access is limited.

### Biopsy-Naïve Patients

Biopsy-naïve (BN) patients provide the cleanest setting for evaluating microUS as an initial imaging and targeting strategy, and most head-to-head evidence derives from BN cohorts [6, 15, 19, 20, 43, 49]. Hofbauer et al. reported that microUS-targeted biopsy detected 73% (58/79) of csPCa versus 76% (60/79) for mpMRI-targeted biopsy, capturing 97% of the csPCa identified by MRI-targeted biopsy [19]. In a BN-predominant study Avolio et al. showed that microUS-targeted plus systematic biopsy achieved csPCa detection comparable to an mpMRI-based approach (63% versus 62%), with additional improvement when combined with PSAD [55]. 

In a propensity-weighted analysis by Castilho Borges et al. (*n* = 1,119), microUS-guided biopsy demonstrated higher csPCa detection than MRI/US fusion biopsy in the combined approach (45% versus 34%; OR 1.61; *P* < 0.01), with a consistent BN subgroup pattern (49% versus 36%); targeted-only comparisons did not differ significantly [34]. Khajir et al. reported broadly comparable performance between MRI-microUS fusion and MRI-conventional US fusion (53.6% versus 55.3% overall; 56% versus 62% in BN men, *P* = 0.14), with lower detection in prior-negative biopsy subgroups [56]. In multivariable analyses, PI-RADS and, when assessed, PRI-MUS consistently predicted csPCa; however, the effect of biopsy modality differed across studies. In the Castilho Borges cohort, microUS-guided biopsy remained independently associated with csPCa detection alongside PI-RADS 5 lesions, whereas Khajir et al. found that the fusion platform was not independently associated with detection after adjustment for biopsy-naïve status, PSAD, age, and PI-RADS score [34, 56]. 

Several limitations constrain the use of microUS as a first-line strategy in BN patients. Although OPTIMUM demonstrated noninferiority for csPCa detection, all patients underwent biopsy regardless of PRI-MUS or PI-RADS score, so the ability of microUS to safely avoid biopsy is less established compared to mpMRI [20]. Systematic sampling still adds meaningful detection when imaging is negative or discordant: about 9% points of csPCa from systematic-only cores in OPTIMUM, and 22% of csPCa missed by both targeted approaches in the Avolio cohort [20, 54]. Many studies enroll mixed biopsy-history populations with underpowered BN subgroups; performance in larger glands and anterior/transition-zone lesions, optimal PRI-MUS thresholds, and integration with PSAD and prostate volume require further validation [30, 32, 46]. 

MicroUS may simplify the first-line pathway in BN patients, particularly where MRI access or workflow delays are limiting, though mpMRI remains better validated for safe biopsy avoidance. BN-focused studies are needed to define when microUS can safely reduce MRI use without compromising csPCa detection.

### Patients with Negative MRI Findings

Even with optimal mpMRI performance, ~ 15–20% of csPCa remain undetected in patients with PI-RADS ≤ 2, often small, non-index, or aggressive lesions (cribriform/intraductal patterns) with poor diffusion-weighted conspicuity [16, 57, 58]. In patients with persistently high suspicion for PCa despite negative mpMRI findings, microUS has shown additional csPCa yield when added to MRI-based pathways [19, 42]. 

In a prospective study (125 patients with negative mpMRI), microUS-targeted biopsy achieved a sensitivity of 97.1% and an NPV of 96.4% for csPCa. Among patients with negative microUS findings, 22.4% could have avoided biopsy; only one csPCa case (2.9% of csPCa) would have been missed. Detection rates correlated with PRI-MUS score, reaching 71.4% in PRI-MUS 5 lesions. Cribriform and intraductal patterns were identified in 41.2% and 11.8% of detected csPCa cases, respectively [59]. Albers et al. (103 BN men with PI-RADS ≤ 2) found no significant overall difference between microUS-informed and standard TRUS biopsy (25% versus 17%), but PRI-MUS stratification yielded csPCa in 16% of PRI-MUS ≤ 2 versus 42% of PRI-MUS ≥ 3, rising to 60% with PSAD ≥ 0.15 ng/mL² [60]. This is consistent with broader evidence that PSAD adjuncts improve MRI-based risk stratification, with combined models achieving sensitivity up to 90% and specificity up to 86% [44, 45]. Long-term follow-up showed 85.7% of microUS-negative men safely avoided biopsy over a median 39 months, with adjusted hazard ratios 0.17, 0.12, and 0.09 for biopsy-free, cancer-free, and csPCa-free survival, respectively [61]. 

Larger cohorts confirm the pattern. Socarrás et al. (microUS + mpMRI fusion) detected cancer in 43% (15/35) and csPCa in 34% (12/35) of mpMRI-negative patients biopsied for persistent suspicion or positive biomarkers; across the full 194-patient cohort, microUS uniquely identified 12 of 108 lesions (11%) missed by other techniques, 92% of which were clinically significant [49]. Wang et al. showed that microUS-informed systematic cores taken outside MRI-visible lesions detected csPCa in 38% of patients, compared with 8% using MRI-targeted cores alone [62]. 

In this clinical setting microUS may serve as a valuable adjunct for PI-RADS ≤ 2 patients: high NPV supports a rule-out role, and combined microUS–mpMRI sampling maximizes detection.

### Repeat Biopsy Setting

In men with prior negative biopsy and persistent suspicion, csPCa detection is lower than in BN patients but remains clinically meaningful: in the PLUM prior-negative cohort, PCa detection was ~ 20% overall and ~ 5% for PI-RADS 3, versus 38% and 15% in BN men, likely reflecting tumor biology, anterior/transition-zone location, and prior sampling effects [63]. Risk calculators incorporating PSA, PSAD, and prostate volume offer reasonable discrimination (AUC ~ 0.78–0.84) but remain imperfect; PSAD alone has limited sensitivity as a sole rule-out criterion [17, 63–65]. MRI-targeted biopsy alone identifies the highest-grade tumor less frequently than systematic template sampling (37.5% versus 42.1%), with undetected csPCa disproportionately located in the anterior prostate, where mpMRI is least reliable [66, 67]. 

Beatrici et al. (304 men with prior negative biopsy) reported higher microUS sensitivity for csPCa than mpMRI (91.2% versus 77.2%) and higher NPV (66.7% versus 45.8%), with comparable specificity (22.2% versus 24.4%) and PPV (56.4% versus 59.8%); on multivariable analysis, microUS was the only imaging modality independently associated with csPCa detection (aOR 6.58, *P* = 0.035) whereas mpMRI was not (aOR 2.25, *P* = 0.21). These findings suggest that MRI limitations may be more pronounced after a negative biopsy and support combined microUS–mpMRI targeting in persistently suspicious or discordant cases [67]. 

Evidence in this setting remains predominantly retrospective and single-center; the only randomized trial (OPTIMUM) enrolled BN patients exclusively [20]. 68Ga-PSMA PET/CT may serve as an investigational adjunct when both microUS and mpMRI are non-diagnostic, though data derive from small, highly selected cohorts [68]. 

### Active Surveillance

Active surveillance (AS) is the standard of care for low-risk PCa, yet 20–50% patients eventually experience upgrading, reclassification, or definitive treatment, reflecting initial under-grading and under-sampling [9, 53, 69, 70]. mpMRI improves csPCa detection but has limited sensitivity for upgrading and is resource-intensive for longitudinal monitoring, prompting interest in more accessible alternatives [53, 69–72]. 

An early feasibility study of 9 AS patients showed microUS outperformed conventional ultrasound and demonstrated mpMRI concordance [73]. In a prospective cohort of 100 men undergoing confirmatory biopsy one year after ISUP GG 1 diagnosis, Maffei et al. found mpMRI sensitivity (100% versus 94.1%) and NPV (100% versus 88.9%) superior to microUS for detecting upgrading to GG ≥ 2 (microUS missed 2 of 34 upgraded cases); however, targeted cores captured most csPCa on both modalities (73.5% versus 64.7%). A microUS-driven, event-triggered strategy could have avoided confirmatory biopsy in 18% of patients while missing 4 upgrading events, supporting selective biopsy pathways in AS [53]. In a separate cohort of 128 AS patients, PRI-MUS ≥ 3 detected 33/34 (98%) upgraded cases versus 29/34 (85%) for PI-RADS ≥ 3, with PRI-MUS strongly predicting upgrading (OR 15.5); both modalities showed high NPV (97% versus 91%), supporting a potential biopsy-sparing role at low scores [74]. 

In AS, microUS offers office-based use and repeatability suited to longitudinal monitoring, but evidence is limited to small single-center cohorts, warranting larger prospective multicenter validation [53, 74]. 

### Correlation with Prostatectomy Pathology

Most microUS–mpMRI comparisons use tracked biopsy as the histologic reference, but whole-mount (WM) prostatectomy pathology remains the most rigorous standard, despite limitations due to differing imaging-plane orientations, tissue deformation, and the absence of spatial tracking [27, 75]. A recent platform employed MRI as an intermediate reference to co-register microUS, mpMRI, and WM pathology, achieving registration errors of 2.23 ± 0.08 mm (microUS–MRI) and 3.90 ± 0.11 mm (microUS–pathology), which are comparable to those of commercial fusion systems. MicroUS identified a higher proportion of index lesions compared to both prospective (91.7% vs. 73.3%) and retrospective (91.7% vs. 80%) MRI reviews. The false-positive rate for regions of interest (ROIs) was similar to retrospective MRI (38.3% vs. 40.8%) but higher than prospective MRI (38.3% vs. 22.7%) [27]. 

In a 429-patient retrospective cohort, GG upgrading at radical prostatectomy occurred in 18.2% of cases overall, with the highest rate after microUS-only biopsy (40%) and the lowest after MRI–microUS (14.9%) or MRI–TRUS (13.1%) fusion; major upgrading (≥ GG2) occurred in 3.5% of cases. Both fusion strategies significantly outperformed systematic biopsy and did not differ significantly from each other [40]. In a propensity-weighted analysis of a radical prostatectomy subgroup (*n* = 156), microUS-guided biopsy yielded lower upgrading than MRI/US fusion (17% versus 38%), although the difference was not significant for targeted cores only (38% versus 45%) [34]. 

For volumetric assessment, microUS underestimated tumor volume less than the most accurate MRI sequence (DWI: −0.15 versus − 0.26 mL) and provided a more accurate prostate volume estimate (− 3 versus − 6 mL underestimation); MRI detected more lesions overall (71% versus 44%) with comparable peripheral-zone performance (74% versus 71%), and microUS more frequently missed small-volume lesions, differences with practical implications for pre-surgical planning [76]. 

### Local Tumor Staging

Accurate local staging, particularly detection of extraprostatic extension (EPE) and seminal vesicle invasion (SVI), guides nerve-sparing, margin status, and adjuvant therapies [77]. mpMRI remains the reference standard but is limited: pooled sensitivity ~ 57% for EPE (specificity ~ 91%) and ~ 58% for SVI (specificity ~ 96%), with stage T3 detection at sensitivity ~ 61% and specificity ~ 88%; accuracy improves when combined with clinical variables (e.g., GG ≥ 3 strongly predicts SVI, while GG < 3 nearly excludes it, NPV 98.5%) [77–79]. 

MicroUS adds real-time capsular visualization for pre- and intraoperative assessment of tumor extension. In a prospective study, four capsular features (capsular bulging, visible extracapsular extension (ECE), hypoechoic halo, and obliteration of the vesicle-prostatic angle) were evaluated; ECE alone predicted EPE with sensitivity 72.1%, specificity 88%, and AUC 80.4%. Incorporating microUS capsular features with clinical parameters increased the model AUC to 87.6%, and non–organ-confined disease was observed in 92.3% of patients exhibiting all four predictors [80]. An earlier feasibility study confirmed sensitivity 87.5% and specificity 80.0% for EPE prediction [81]. In a side-specific analysis (*n* = 391, 612 lobes), a microUS-based nomogram incorporating PSAD, ISUP grade group, maximal core involvement, and microUS-detected ECE achieved internally validated AUC 0.81, comparable to an mpMRI model while identifying a substantial proportion of MRI-missed EPE [82]. For apical cancer, microUS sensitivity reached 81%, comparable to PSMA-PET and superior to MRI (66%), but with lower specificity (14%); combined multimodal assessment reduced missed apical cancers to 1% in a 619-patient robot-assisted radical prostatectomy (RARP) cohort [83]. MicroUS is also emerging for EPE-guided margin decisions and focal-therapy guidance, with intraoperative nerve-sparing applications under prospective evaluation [84, 85]. 

Evidence for SVI assessment remains limited, and a highly operator-dependent workflow currently favors mpMRI for definitive pre-treatment staging; microUS offers complementary real-time capsular assessment and emerging intraoperative value.

## Limitations

### Technical and Implementation Limitations

A key limitation of microUS is the limited penetration of its 29-MHz frequency, which can compromise visualization in larger prostates and anterior or transition-zone lesions, where missed csPCa is repeatedly reported [19, 86]. Lacking functional sequences (DWI/DCE), microUS has higher false-positive rates than mpMRI; a meta-analysis of five prospective studies reported pooled sensitivity 0.84 and specificity 0.41, supporting a triage or rule-out role while highlighting the need for standardized PRI-MUS thresholds, as no validated cutoff exists for safe biopsy avoidance comparable to PI-RADS ≤ 2 [6, 20, 22]. MicroUS also more frequently misses small-volume (≤ 0.1 mL) and GG1–2 lesions, which is important in treatment planning and AS follow-up [76]. Most comparative evidence derives from retrospective, single-center cohorts with heterogeneous biopsy histories and limited blinding; broader adoption will require multicenter validation and remains constrained by capital costs, single-vendor reliance, and the absence of standardization comparable to PI-RADS [11, 87]. 

### Inter-reader Agreement and Reproducibility

PRI-MUS scoring is performed in real time during biopsy, unlike mpMRI’s archival interpretation, which causes increasing inter-reader variability and limits opportunities for delayed expert review [6, 11, 15]. In a multicenter study (six urologists), agreement was only fair (κ = 0.30) with patient-level csPCa sensitivity 0.66 (lesion-level 0.58); combined reader annotations improved performance to 0.85 (patient) and 0.77 (lesion), suggesting a role for collaborative or AI-assisted interpretation. Anterior tumors were disproportionately missed (up to 62%), and reproducibility remains inferior to mpMRI (κ 0.41–0.59) [88–90]. 

### Learning Curve and Operator Dependence

MicroUS performance is highly operator-dependent, and proficiency is not immediate. Comparable mpMRI thresholds are ~ 40 cases for PI-RADS proficiency and 100–110 for MRI–TRUS fusion biopsy [87, 91–93]. Operators with prior fusion experience achieve expert sensitivity after 20–40 microUS cases and expert specificity after 40–90 with structured training, with ~ 3 h of simulator practice for needle localization [48, 94]. In an early RCT of first-generation microUS, mid-course training increased csPCa detection from 32% to 39% [95]. OPTIMUM mandated “Advanced” certification (≥ 50 cases) for all operators (12 reached “Expert,” ≥90 cases) to mitigate learning-curve effects [20]. Unlike MRI workflows, microUS combines real-time interpretation and biopsy in a single operator, underscoring the need for standardized training and decision-support tools [15, 20]. 

## Future Directions

MicroUS is advancing toward an integrated diagnostic–therapeutic platform through parallel developments in AI and image-guided intervention. AI is designed to reduce operator dependence and enable real-time decision support [96, 97]. Current models achieve AUC ~ 0.75 with lower sensitivity (~ 68%) than PI-RADS or PRI-MUS (88–94%) but higher specificity (~ 70% versus 22–45%) and comparable overall accuracy, outperforming novice readers and approaching expert level [96]. Generalizability remains limited by operator-dependent acquisition, imaging artifacts, and small training datasets. Many studies also rely on cognitive rather than precise spatial co-registration with pathology, weakening the histologic reference standard [96, 98]. Recent AI work in microUS includes uncertainty-aware detection frameworks such as TRUSWorthy, segmentation models such as MicroSegNet, and cancer-detection architectures such as TRUSformer, ProstNFound, and ProMUS-NET; in head-to-head evaluations, several have matched or exceeded expert urologist performance [89, 99–102]. In clinical practice, AI is expected to be deployed as real-time probabilistic heatmaps that guide targeted biopsy while preserving systematic sampling, although prospective validation and standardized annotation frameworks remain necessary [96]. 

MicroUS supports real-time needle placement, tissue-change monitoring, and intra-procedural margin assessment across focal therapy modalities (cryoablation, laser ablation, HIFU); integrated with mpMRI for initial risk stratification, it compensates for a key MRI limitation: systematic underestimation of tumor extent where margin precision is critical [27, 76, 103, 104]. Emerging hardware such as transurethral microUS via cystoscope and dual-frequency probes may improve penetration but remains experimental [87]. 

## Conclusions

MicroUS has emerged as a high-resolution imaging modality with csPCa diagnostic performance comparable to mpMRI, offering practical advantages in accessibility, cost, and real-time workflow integration. Combined mpMRI/microUS approaches yield the highest detection accuracy, positioning microUS as a complement rather than a replacement for mpMRI at this stage. With continued prospective validation and standardization of PRI-MUS interpretation, microUS is well-positioned to evolve from a biopsy-guidance tool into a unified platform spanning detection, real-time targeting, and treatment planning. Advances in AI-assisted interpretation and image-guided focal therapy hold promise for improving reproducibility and enabling precision-oriented prostate cancer care.

## Data Availability

No datasets were generated or analysed during the current study.
